# Bowling: Occupational Hazards of the Wrist and Hand in Elite Tenpin Bowlers

**DOI:** 10.5704/MOJ.2103.017

**Published:** 2021-03

**Authors:** YJ Lee, TCY Harmony, IS Jamal-Azmi, J Gunasagaran, TS Ahmad

**Affiliations:** 1 Department of Orthopaedic Surgery, University of Malaya, Kuala Lumpur, Malaysia; 2 Department of Anaesthesiology, University of Malaya, Kuala Lumpur, Malaysia

**Keywords:** sports injuries, athletes, tendinopathies, deQuervain’s tenosynovitis, kinematics

## Abstract

**Introduction:**

Bowling is an immensely popular, but scarcely researched sport associated with overuse injuries in its participants. The purpose of this study was to investigate and report on the incidence of common upper extremity complaints in elite bowling athletes.

**Materials and methods:**

All Malaysian national level bowlers (n=39) were evaluated via questionnaire on their upper limb symptoms. A focused, relevant clinical examination was performed on each subject to exclude de Quervain’s tenosynovitis, tennis and golfer's elbow, carpal tunnel syndrome and trigger finger. The athletes were then allowed to resume bowling for two hours before completing another symptom-related questionnaire.

**Results:**

Pain was the predominantly observed symptom, with a predilection for the wrist, ring and middle fingers, and thumb. De Quervain’s tenosynovitis was found in 53.8% (n=21) of the subjects, with 52.4% and 42.9% of them experiencing pain during and after training, respectively. Other repetitive injury-related disorders were also considerably more common than in their non-playing limb and the general population.

**Conclusion:**

The incidence of de Quervain’s tenosynovitis was exceptionally high in this population. Further studies on sports kinematics are needed to prevent long term morbidities in these athletes.

## Introduction

In a game of tenpin bowling, a weighted ball that is gripped using only the thumb, middle and ring fingers, is swung and subsequently launched at speed along an 18m long alley, in an attempt to knock down as many of the pins as possible, thus accumulating as many points as possible. When performed repetitively, these movements could result in overuse injuries in the elbows, wrists and hands of its participants. As well known as the sport is, and despite the tremendous amount of research dedicated to the field of sports medicine, little effort has been devoted to investigating the ill effects of this sport on the musculoskeletal health of its players.

Approximately 3% to 25% of sports related injuries were reported to involve the wrist and hand^1-3^. Based upon the kinematics of tenpin bowling as a throwing sport, the game places its participants at risk of these musculoskeletal disorders. However, literature describing the relationship of competitive bowling to musculoskeletal ailments has, to our best knowledge, been few and far between. An example of this is an article by Kerr *et al* in 2011^4^. The epidemiological study described bowling related injuries in patients presenting to the emergency department in 18 years. Finger injuries were the commonest predicament, followed by injuries to the trunk and the wrist. Details of these injuries were, however, vague and non-specific; they were mainly attributed to 'soft tissue injuries' and 'sprains or strains'. Furthermore, the commonest mechanisms of injuries described in the article, of tripping and falling, or being hit by the bowling ball, and injuries as a result of dropping the ball, are less likely to occur in more seasoned athletes and do not represent or occur as a result of, repetitive stress^4^.

This study was carried out to investigate, describe and report the incidence and symptomatology of common upper limb pathology in elite bowling athletes.

## Material and Method

This observational study was reported according to the STROBE guidelines. A universal sampling technique was performed: all present Malaysian National Elite Team and National Back-Up Bowling Team, coached under the same standard national elite training programme, were recruited. The bowlers were initially cross-sectionally interviewed via a questionnaire to ascertain their demographic information, bowling styles, bowling history and upper limb musculoskeletal symptoms.

Parameters to determine each athlete's characteristic bowling style included their handedness and bowling handedness, preferred bowling ball weight and ball gripping methods. The weights of bowling balls were classified as light (4.5 kg to 5.0 kg), medium (5.1 kg to 6.0 kg), or heavy (6.1 kg to 7.0 kg). Ball gripping methods, on the other hand, were categorised either as 'conventional', where the ball is gripped up to the proximal interphalangeal joint or as 'fingertip', where it is gripped up to only the distal interphalangeal joint.

The total period of active bowling for each bowler was recorded in the bowling history and was defined as the accrued duration, in years, spent training as a national athlete. The number of sessions of bowling and the average number of hours per session were also noted. The cumulative hours of bowling, defined as the total number of hours spent on bowling, was then derived from this data. The presence of previous and/or current upper limb musculoskeletal symptoms throughout their bowling career and the specific nature and duration of each symptom, viz. subjective pain, swelling and stiffness, were subsequently noted for both elbows and wrists and the digits of each hand.

Next, a targeted clinical examination of both the upper limbs of each athlete was carried out. Tests performed were: the Finkelstein, Eichhoff and the wrist hyperflexion and abduction of the thumb (WHAT) tests for the diagnosis of de Quervain’s tenosynovitis; the Durkan test, Tinel sign, and numbness in the median nerve distribution in the digits for the diagnosis of the carpal tunnel syndrome; tenderness along with the A1 pulley and observed triggering of the digit for the diagnosis of a trigger finger; the Mill and Cozen tests, and common extensor origin tenderness for the diagnosis of a tennis elbow; and common flexor origin tenderness and pain on passive wrist flexion and forearm pronation for the diagnosis of a golfer's elbow. Each set of tests was performed only when the relevant symptoms were registered on the questionnaire. The Finkelstein, Eichhoff and WHAT tests were only performed when the subject had symptoms of wrist pain. A clinical diagnosis was made if at least one of the tests was positive for each respective pathology.

The athletes were then allowed to resume bowling for two hours of their routine training sessions before another questionnaire-based evaluation of their symptoms of pain if any, was conducted. The presence of pre-game, in-game and post-game pain and their respective pain scores were recorded for the wrist and elbow pathologies of de Quervain's tenosynovitis, tennis and golfer's elbow.

Data collection was led by a senior orthopaedic hand surgeon, and every member of the team was well-versed in the techniques of clinical examination of the hand and upper limb. All questionnaire and clinical examination data were transcribed and analysed using the SPSS version 25 software. Descriptive statistics were used to report demographic data and incidence of musculoskeletal symptoms. The Fisher’s exact test was used for comparison of symptoms between playing and non-playing limbs.

This study was approved by our institutional ethics committee (Ethics Approval Number: MREC ID NO: 201941-7277).

## Results

There were 39 members in the present Malaysian National Elite Team and National Back-Up Bowling Squad, and all were recruited as subjects Table I. Twenty-one (53.8%) of them were women. The mean age of the athletes was 24.0 years old, with a range of 18 - 42 years. Predictably, a majority were right-handed (87.2%, n=34) and consequently, the majority bowled with their right hands (87.2%, n=34); however, two right-handed athletes bowled with their left, and another two left-handed ones bowled with their right. Two-thirds (74.4%, n=29) of the athletes bowled with a fingertip grip, and all of them (n=39) utilised heavy bowling balls. The mean total period of active bowling for the athletes was 13.4 years (range, 4-31). They bowled for a mean of 5 years; 2 sessions per week (range, 5-10) with a mean of 2.3 hours per session (range, 2-7). The mean cumulative hours of bowling was 5281.9 hours (range, 384-22080) Table I.

**Table I:** Demographic data of bowlers.

Categorical demographic data	n	%
Gender		
Male	18	46.2%
Female	21	53.8%
Handedness		
Right	34	87.2%
Left	5	12.8%
Ball gripping handedness		
Right	34	87.2%
Left	5	12.8%
Method of ball gripping		
Fingertip	29	74.4%
Conventional	10	25.6%

Categorical demographic data	Mean	Standard Error	Range	
			Minimum	Maximum
Age (years)	24.0	0.9	18.0	42.0
Period of most active bowling (years)	9.5	1.0	0.8	31.0
Total period of active bowling (years)	13.4	0.9	4.0	31.0
Sessions per week	5.2	0.1	5.0	10.0
Hours per session (hours)	2.3	0.2	2.0	7.0
Cumulative hours of bowling (hours)	5281.9	667.9	384.0	22080.0

In terms of topographical involvement (Table II(A) and II(B)), the wrist (66.7%, n=26), ring finger (43.6%, n=17), middle finger (30.8%, n=12) and thumb (20.5%, n=8) of the ball-playing limb were the four commonest structures which were previously and/or currently symptomatic, on enquiry. More specifically, pain was the predominant symptom in each of these: wrist pain 59.0%, n=23; ring finger pain 28.2%, n=11; middle finger pain 15.4% n=6; thumb pain 17.9%, n=7. In juxtaposition, symptoms in these structures in the contralateral non-playing limb were minimal: only one subject complained of wrist symptoms, swelling, stiffness and pain; and another complained of elbow pain; whilst none complained of any digital symptoms in the non-playing limb.

**Table II(a):** The incidence of previous and/or current upper limb symptoms in ball-playing limb of the study subject

Symptoms	n	Ball-playing Limb %	95% CI
Wrist symptoms	26	66.7	(49.8, 80.9)
Thumb symptoms	8	20.5	(9.3, 36.5)
Index finger symptoms	0	0	(0, 9.0)
Middle finger symptoms	12	30.8	(17.0, 47.6)
Ring finger symptoms	17	43.6	(27.8, 60.4)
Little finger symptoms	0	0	(0, 9.0)
Elbow symptoms	3	7.7	(1.6, 20.9)

**Table II(b):** Comparison of upper limb symptoms between ball-playing limbs and non-playing limbs

Symptoms	Ball-playing limb		Non-playing limb		Fisher Exact Test
	n	%	n	%	(p- value)
Wrist symptoms	26	66.7	1	2.6	< 0.01
swelling	4	10.3	1	2.6	0.358
stiffness	7	17.9	1	2.6	0.056
pain	23	59	1	2.6	< 0.01
Thumb symptoms	8	20.5	0	0	< 0.01
swelling	5	12.8	0	0	0.055
stiffness	1	2.6	0	0	1
pain	7	17.9	0	0	0.012
Middle finger symptoms	12	30.8	0	0	< 0.01
swelling	3	7.7	0	0	0.24
stiffness	3	7.7	0	0	0.24
pain	6	15.4	0	0	0.025
Ring finger symptoms	17	43.6	0	0	< 0.01
swelling	3	7.7	0	0	0.24
stiffness	3	7.7	0	0	0.24
pain	11	28.2	0	0	< 0.01
Elbow symptoms	3	7.7	1	2.6	0.615

In the ball-playing limb, wrist pain was reported to have lasted between one to 6 months in 10 (25.6%) subjects and beyond 12 months in only one (2.6%). Thumb and middle finger pain were found to have lasted between one to 6 months in one subject and beyond 12 months in another 5. Ring finger pain was reported to have lasted between one to 6 months and more than 12 months in 2 and 6 subjects, respectively. When a subsequence analysis was performed to evaluate the correlation between the two ball gripping methods Table III, it was observed that athletes who bowled with the ‘conventional’ grip were more likely to be symptomatic (100%; 10 out of 10 subjects) when compared to those who bowled with the ‘fingertip’ grip (86.2%; 25 out of 29 subjects). However, the difference was not statistically significant.

**Table III:** The incidence of symptomatic bowlers and the associated upper limb symptoms grouped according to their preferred ball gripping method

	Fingertip		Conventional		Fisher Exact Test
	n	%	n	%	(p- value)
Symptomatic bowler	25	86.2	10	100	0.556
Wrist	19	65.5	7	70	1
Thumb	6	20.7	2	20	1
Middle Finger	9	31	3	30	1
Ring Finger	13	44.8	4	40	1
Elbow	2	6.9	1	10	1

In the targeted examination section Table IV, the commonest diagnosis made in the athletes’ ball-playing limb was de Quervain's tenosynovitis; it was diagnosed in a remarkable 53.8% (n=21) of these athletes. Tennis elbow and golfer's elbow were found in 3 (7.7%) and 2 (5.1%) subjects, respectively, while a diagnosis of trigger thumb, index and ring finger trigger fingers were made in 2, one, and 3 of them, respectively. By comparison, in the non-playing limb, only one out of the 39 athletes was diagnosed with a tennis elbow, and none fulfilled the diagnostic criteria for any of the other conditions. When the bowlers were reevaluated after their session of training, 11 of the 21 (52.4%) athletes diagnosed with de Quervain’s tenosynovitis were found to have wrist pain in their ball-playing limb during training with a mean pain score of 3.0 (on a scale of 0 to 10), and 9 (42.9%) were found to have post-game wrist pain with a mean pain score of 2.38. None of those diagnosed with elbow conditions (tennis and golfer's elbow) complained of pain during or after their two-hour game.

**Table IV:** The incidence of specific upper limb musculoskeletal conditions diagnosed through a targeted physical examination

Diagnosis		Ball-playing Limb	
	n	%	95% CI
De Quervain's tenosynovitis	21	53.8	(37.2, 69.9)
Trigger thumb	2	5.1	(0.6, 17.3)
Trigger Index Finger	0	0	(0, 9.0)
Trigger Middle Finger	3	39	(1.6, 20.9)
Trigger Ring Finger	5	12.8	(4.3, 27.4)
Trigger Little Finger	0	0	(0, 9.0)
Carpal Tunnel Syndrome	2	5.1	(0.6, 17.3)
Tennis Elbow	3	7.7	(1.6, 20.9)
Golfer's Elbow	2	5.1	(0.6, 17.3)

## Discussion

Malaysian Elite tenpin bowlers suffer from pain involving the wrist, ring finger, middle finger and thumb of the ball-playing limb. This was frequently accompanied by symptoms of stiffness and swelling of these parts of the body. Those complaining of wrist symptoms were found to have de Quervain’s tenosynovitis 80% of the time, which was the commonest diagnosis on clinical examination, with an incidence of 54%. This incidence is considerably higher than in the average population; only 0.49% of the population were reportedly affected by de Quervain’s tenosynovitis in one recent study^5^, and even so, it was more routinely found in older age groups^5,6^. Other diagnoses of tennis and golfer's elbow, carpal tunnel syndrome and trigger finger were less prevalent but were still much more common when compared to the non-playing limb. The incidence of these disorders was also higher in comparison to the average population^5,7-9^.

These findings can quite clearly be attributed to a few aspects of how the game is played. In order to produce a greater ‘pin action’ to generate a domino effect on the other bowling pins, players nearly always attempt a ‘hook’ to create a spin on the bowling ball^10^; this requires releasing the ball exclusively via a wrist action, which increases the work and hence repetitive strain around it. Another obvious contributing factor would be the awkward and unnatural ball-gripping technique involved, where only the thumb, middle and ring fingers are fitted within the gripping holes of a ball; the statistics in this study where many of the subjects recorded symptoms in their thumbs, middle and ring fingers, and where none of them recorded any symptoms in the index and little fingers substantiate this. The 'conventional grip' method where the ball is gripped up to the proximal interphalangeal joint by which individual bowlers prefer to grip their ball, was also found to be associated with slightly more frequent symptoms here. Although a similar comparison cannot be made in the subjects here, in view that all of them utilised the same weight class of balls, it can be asserted that the use of a heavier bowling ball would likewise increase the risk of musculoskeletal afflictions due to the increase in force required to handle and launch them.

Another factor that should be taken into account would be the amount of time these athletes spent in training; most were observed to spend an average of two and a half hours per practice session five times a week. This may seem a fair amount given that bowling may be their primary priority as professionals, but such high frequencies of training could also precipitate an injury or pathology and/or prevent its healing^11,12^. This was reflected by the fact that a good number of these athletes bowled on, despite the awareness of their symptoms, some lasting for longer than 12 months, and that a few of them even continued training despite experiencing pain before, during and/or after their game.

These musculoskeletal disorders may ultimately result in poorer performance, days of work lost^13,14^, long term morbidities or may even end careers if left untreated. It is therefore paramount that initiatives are taken to identify, diagnose and treat them at an early stage as these conditions are known to respond favourably to both non-operative and operative management. Awareness programmes, more personalised training schedules and modern training methods, an improvement in throwing technique, and the development of highly individualised equipment may help retard or deter their occurrence. The area of further research should be focused on assisting in the formulation of other preventive strategies and to improve the athletes' performances whilst protecting them from trauma.

This study is not without limitations. The small sample size of 39 subjects precludes more complex statistical analyses and thereby the ability to arrive at more reliable and credible conclusions. This was however unavoidable as the subjects of this study constitute the entirety of the national bowling squad bowling at the highest level in our country. The questionnaire data heavily depended on the comprehension and mental recollection abilities of the subjects, and therefore may not be precise. The deployment of an experienced team to the task of data collection and of performing the targeted clinical examination may have reduced but not eliminated the possibility of data reporting errors. Lastly, many other common overuse injuries and conditions were not investigated within this observational study; an example of which is hand osteoarthritis, which was initially planned as a part of this report but only to be excluded due to the lack of radiographic support for diagnostic confirmation.

## Conclusion

Elite tenpin bowling athletes are at an increased risk of developing musculoskeletal disorders of the upper limb. The incidence of de Quervain’s tenosynovitis was exceptionally high in this population. The pathogenesis of these disorders may be linked to the kinematics of the sport and should be investigated further to improve the performances and prevent long term morbidities in these athletes.

## References

[1] Avery DM, Rodner CM, Edgar CM (2016). Sports-related wrist and hand injuries: a review.. J Orthop Surg Res..

[2] Rosenbaum YA, Awan HM (2017). Acute hand injuries in athletes.. Phys Sportsmed..

[3] Shariff AH, George J, Ramlan AA (2009). Musculoskeletal injuries among Malaysian badminton players.. Singapore Med J..

[4] Kerr ZY, Collins CL, Comstock RD (2011). Epidemiology of bowling-related injuries presenting to US emergency departments, 19902008.. Clin Pediatr (Phila)..

[5] Shen PC, Chang PC, Jou IM, Chen CH, Lee FH, Hsieh JL (2019). Hand tendinopathy risk factors in Taiwan: A population-based cohort study.. Medicine (Baltimore)..

[6] Wolf JM, Sturdivant RX, Owens BD (2009). Incidence of de Quervain's tenosynovitis in a young, active population.. J Hand Surg Am..

[7] Descatha A, Leclerc A, Chastang JF, Roquelaure Y (2003). Medial epicondylitis in occupational settings: prevalence, incidence and associated risk factors.. J Occup Environ Med..

[8] Shiri R, Viikari-Juntura E, Varonen H, Heliovaara M (2006). Prevalence and determinants of lateral and medial epicondylitis: a population study.. Am J Epidemiol..

[9] Smidt N, van der Windt DAWM (2006). Tennis elbow in primary care. BMJ..

[10] Frohlich C (2004). What makes bowling balls hook?. Am J Phys..

[11] Dennis RJ, Finch CF, Farhart PJ (2005). Is bowling workload a risk factor for injury to Australian junior cricket fast bowlers?. Br J Sports Med..

[12] Jones CM, Griffiths PC, Mellalieu SD (2017). Training Load and Fatigue Marker Associations with Injury and Illness: A Systematic Review of Longitudinal Studies.. Sports Med..

[13] Thompson AR, Plewes LW, Shaw EG (1951). Peritendinitis crepitans and simple tenosynovitis; a clinical study of 544 cases in industry.. Bri J Ind Med..

[14] Kurppa K, Viikari-Juntura E, Kuosma E, Huuskonen M, Kivi P (1991). Incidence of tenosynovitis or peritendinitis and epicondylitis in a meat-processing factory.. Scand J Work Environ Health..

